# Parallelized short read assembly of large genomes using de Bruijn graphs

**DOI:** 10.1186/1471-2105-12-354

**Published:** 2011-08-25

**Authors:** Yongchao Liu, Bertil Schmidt, Douglas L Maskell

**Affiliations:** 1 School of Computer Engineering, Nanyang Technological University, Singapore; 2 Institut für Informatik, Johannes Gutenberg University Mainz, Germany

## Abstract

**Background:**

Next-generation sequencing technologies have given rise to the explosive increase in DNA sequencing throughput, and have promoted the recent development of *de novo *short read assemblers. However, existing assemblers require high execution times and a large amount of compute resources to assemble large genomes from quantities of short reads.

**Results:**

We present PASHA, a parallelized short read assembler using de Bruijn graphs, which takes advantage of hybrid computing architectures consisting of both shared-memory multi-core CPUs and distributed-memory compute clusters to gain efficiency and scalability. Evaluation using three small-scale real paired-end datasets shows that PASHA is able to produce more contiguous high-quality assemblies in shorter time compared to three leading assemblers: Velvet, ABySS and SOAPdenovo. PASHA's scalability for large genome datasets is demonstrated with human genome assembly. Compared to ABySS, PASHA achieves competitive assembly quality with faster execution speed on the same compute resources, yielding an NG50 *contig *size of 503 with the longest correct *contig *size of 18,252, and an NG50 *scaffold *size of 2,294. Moreover, the human assembly is completed in about 21 hours with only modest compute resources.

**Conclusions:**

Developing parallel assemblers for large genomes has been garnering significant research efforts due to the explosive size growth of high-throughput short read datasets. By employing hybrid parallelism consisting of multi-threading on multi-core CPUs and message passing on compute clusters, PASHA is able to assemble the human genome with high quality and in reasonable time using modest compute resources.

## Background

The emergence and widespread adoption of massively parallel next-generation sequencing technologies has given rise to the explosive increase in DNA sequencing throughput at a substantially lower unit cost of data, compared to conventional Sanger capillary-based technologies. However, these technologies introduce some new challenges to the assembly of large genomes due to two factors: (i) short read length and (ii) high throughput. This poses a challenge to the bioinformatics community to devise assembly software that can deal with a massive amount of short reads in reasonable time using modest and accessible compute resources.

Consequently, several assemblers for high-throughput short reads have been recently developed. They can be classified into two approaches: *contig *extension and de Bruijn graph. The *contig *extension approach is based on the base-by-base extension at the 3' end of a *contig *sequence by finding overlaps between the prefixes of reads and the suffixes of the *contig*. SSAKE [1], VCAKE [2], SHARCGS [3], Taipan [4], and PE-assembler [5] are example assemblers using this approach. The de Bruijn graph approach to assembly was first introduced in Pevzner et al. [6], and several short read assemblers based on de Bruijn graphs have been developed. Prominent examples include ALLPATHS [7], Velvet [8], ABySS [9] and SOAPdenovo [10]. Due to the enormous cost in terms of both memory and execution time, ALLPATHS was initially constrained to the assembly of small genomes and recently has been extended to support large genomes [11]. Velvet employs a bi-directed simplified de Bruijn graph data structure, which requires accommodating the entire genome in the graph, resulting in a large amount of memory consumption for large genomes. Furthermore, when joining *contigs *into *scaffolds *using paired-end data, Velvet stores the read mapping locations and paired-end information along with the graph, making it infeasible for assembling large genomes. ABySS employs a distributed de Bruijn graph data structure. It is implemented using the message passing interface (MPI), and produces *contigs *in parallel on a distributed-memory compute cluster without the use of paired-end information. SOAPdenovo employs a de Brujin graph data structure similar to that of Velvet, but uses a multi-threaded design to parallelize compute-intensive portions on shared-memory architectures. Besides those algorithms that use directed de Brujin graphs, YAGA [12] employs a bi-directed string graph, represented by a set of edges, and produces *contigs *through path walking using a variation of the classic parallel list ranking problem. This algorithm shows good parallel scalability for small microbial genomes. However, to assemble the E.coli dataset (see the Results and Discussion section), its execution time (496 seconds) using 256 CPUs of a Blue Gene/L system is longer than PASHA (325 seconds) on a single CPU core (see the Results and Discussion section).

In this paper, we present PASHA, a parallelized short read assembler for large genomes based on de Bruijn graphs. Some of the concepts adopted in PASHA are inspired by Velvet and ABySS. The primary contribution of our algorithm is the usage of hybrid parallelism consisting of small-scale shared-memory multi-threading on multi-core CPUs and large-scale distributed-memory parallelism on compute clusters to overcome memory constraints and to achieve high speed for large genome assembly. Furthermore, we incorporate several techniques (e.g. a modified Tour-bus method [8] to remove bubbles and a modified Pebble algorithm [13] to join *contigs*) in the typical assembly pipeline to facilitate the improvement of assembly quality. Evaluation using three small-scale real paired-end datasets indicates that PASHA produces higher-quality assemblies than Velvet, ABySS and SOAPdenovo in less time. To demonstrate the capability of assembling large genomes, we assembled 3.76 billion paired-end short reads from the whole-genome sequencing of a Yoruban male individual (NA18507) from Bentley et al. [14], and obtained an NG50 *contig *size of 503, with the longest correct *contig *size of 18,252, and an NG50 *scaffold *size of 2,294.

The size of the assembly problem severely impacts on the assembly algorithm. The PASHA and ABySS assemblers are implemented using MPI and are able to run on both shared-memory and distributed memory computer clusters. SOAPdenovo is designed using multi-threading and thus is only suitable for shared-memory computers. Because SOAPdenovo requires a computer system with a large amount of shared memory (around 512 GB for this assembly problem), we are not able to execute it to assemble the Yoruban male individual genome on the hardware resources available to us (a symmetric multiprocessing server with 48 CPU cores and 256 GB memory) and thus exclude it from the comparison. For the complete assembly, PASHA took about 21 hours using only modest computing resources, achieving competitive assembly quality with faster execution speed, compared to ABySS.

## Methods

Even though de Bruijn graph-based assemblers successfully alleviate the pressure on memory space and execution speed by substituting reads with *k*-mers (a contiguous sequence of *k *bases) as nodes, compared to the conventional overlap-layout-consensus approaches, the memory consumption and execution time is still prohibitive for large genomes. For example, for the genomic data of a Yoruban male individual, the total number of nodes in the preliminary de Bruijn graph (a node corresponds to a distinct 27-mer) is about 7.73 billion. This motivates us to design a scalable assembler for large genomes that is workable on modest and commonly used high-performance computing resources.

**Figure 1:**
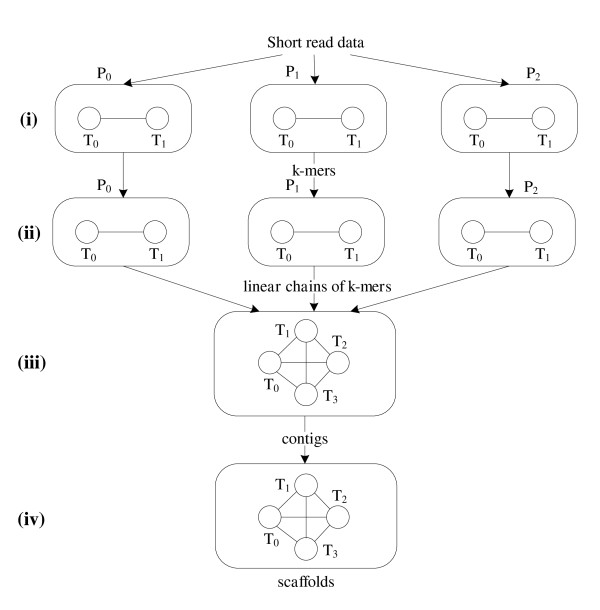
**Schematic diagram of the PASHA assembly pipeline**. (i) k-mer generation and distribution over a number of MPI processes; (ii) distributed preliminary de Bruijn graph construction and simplification over a number of MPI processes; (iii) Bubble merging and contig generation; and (iv) scaffolding.

PASHA is a parallelized algorithm for large genome assembly, which overcomes the memory and execution speed constraints by using hybrid computing architectures consisting of shared-memory multi-core CPUs and distributed-memory compute clusters. Figure 1 illustrates the pipeline of our assembler. The pipeline comprises four stages: (i) generating and distributing *k*-mers, (ii) constructing and simplifying the distributed preliminary de Bruijn graph, (iii) merging bubbles and generating *contigs *after constructing a Velvet-like de Bruijn graph, and (iv) scaffolding to join *contigs *into scaffolds. We have implemented Stages (i) and (ii), which are suitable for parallelization and the most memory-intensive, using MPI. This makes our program compatible with both shared-memory and distributed-memory computing systems. Each MPI process *P*_*i *_comprises two threads *T*_0 _and *T*_1_. *T*_0 _performs computations for the assembly pipeline, and *T*_1 _performs communications between different processes (see (i) and (ii) in Figure 1), as well as file I/O operations. By employing two threads in a single process, we intend to gain faster speed by overlapping the local computation and remote communications with processes (and file I/O). By distributing the de Bruijn graph over a network of computers, we get a partition of the graph with each part stored in a different computer. Hence, we do not need a large amount of memory in a single computer, making our algorithm workable even on a compute cluster comprised of commonplace workstations or personal computers. Since the size of a message is very small, sending messages one-by-one to remote processes will incur large communication overheads. Thus, for the messages that are not time-critical, we combine them into packets to improve the communication efficiency. *T*_0 _and *T*_1 _are connected through a bi-directional message queue. The maximal number of messages in the queue is controlled by a maximal capability threshold. Any thread, which tries to append a new message to the queue, will be blocked if the queue reaches the threshold, and will be resumed once the queue has spaces available. Any thread that tries to retrieve a message from an empty queue will be blocked until there is at least one message available. For Stages (iii) and (iv), which exhibit limited parallelism and are less memory-intensive, we use a multi-threaded design, only compatible with shared-memory systems.

In our proof-of-concept implementation (the source code is available for download at http://sites.google.com/site/yongchaosoftware/pasha), we have used parts of the source code from Velvet for Stages (iii) and (iv) with some algorithmic and data structure modifications. The use of existing open-source code significantly reduces the development time for prototyping our algorithm, and more importantly, our modifications make the two stages feasible and practical to execute on a workstation with limited system memory (i.e. 72 GB RAM in our workstation), as well as providing better assembly qualities. PASHA supports the standard FASTA and FASTQ input formats for single-end and paired-end short reads with different insert sizes. While some other assemblers require users to tune a number of parameters to gain the best assembly, PASHA only needs a single parameter "-k" (i.e. the *k*-mer size), making it relatively user friendly.

Before describing PASHA in details, we firstly define some terms to facilitate our discussion. Given a sequence *S *of length *l*, we define *S*[*i*] as the *i*-th base in the sequence, $\bar{S\left[i\right]}$ as the complement of *S*[*i*], *S*_*i *_as the *k*-mer starting at position *i *(1≤*i*≤*l*-*k*+1) of *S*, $\bar{S_{i}}$ as the reverse complement of *S*_*i*_, and $S_{i}^{c}$ as the canonical *k*-mer that is the lexicographically smaller of *S*_*i *_and $\bar{S_{i}}$. A *k*-molecule of *S*_*i *_is a pair of complementary *k*-mer strands consisting of the canonical *k*-mer of *S*_*i *_(i.e. $S_{i}^{c}$) and the non-canonical *k*-mer of *S*_*i *_(i.e. the reverse complement of $S_{i}^{c}$).

### K-mer generation and distribution

In a de Bruijn graph, a node corresponds to a *k*-mer and an edge between two nodes is created if and only if their corresponding *k*-mers have a suffix-prefix overlap of *k*-1 bases. Hence, PASHA starts the construction of its preliminary de Bruijn graph from the generation of all *k*-mers from the input read data.

As mentioned above, PASHA employs an MPI-based approach to *k*-mer generation and distributes them among the processes. This distribution requires that the location of any *k*-mer is deterministic and can be efficiently computed from the *k*-mer itself. Since a *k*-molecule is a pair of complementary strands, the location of a *k*-mer and its reverse complement must be the same. Before calculating the location of *S*_*i *_and $\bar{S_{i}}$, PASHA first transforms *S*_*i *_and $\bar{S_{i}}$ to their corresponding base-4 representation by assigning numerical value {0, 1, 2, 3} to bases {A, C, G, T}. To determine the location of *S*_*i*_, unlike ABySS (which calculates the hash values of *S*_*i *_and $\bar{S_{i}}$, and then performs an XOR operation on them), PASHA computes the location from the canonical *k*-mer. Since the base-4 representation of a *k*-mer is stored in a 64-bit integer (thus limiting the maximum allowable *k*-mer size to 31), the comparison can be theoretically completed in one clock cycle on a 64-bit computing system. A balanced distribution of *k*-mers among processes is critical to the performance of our algorithm in terms of both execution time and memory space. An unbalanced distribution would cause some processes to consume much more memory for *k*-mer storage, thus resulting in a system failure due to memory limitations on some compute nodes. In PASHA, we first calculate a hash value *I*_*k *_using a linear congruential hash function from the base-4 presentation of the canonical *k*-mer. Then, the ID of the process that owns this *k*-mer is computed as *I*_*k *_% *N*_*p*_, where *N*_*p *_is the number of processes. From our experiments, our location determination method is able to (roughly) balance the distribution of *k*-mers (e.g., for the Yoruban Male genome assembly using 16 processes, the average number of local 27-mers for each process is 483,194,335 ± 963,003).

To achieve memory efficiency, we use the *sparse_hash_set *template class in the Google Sparse Hash library (http://code.google.com/p/google-sparsehash) to distinguish and store *k*-mers. In PASHA, each process holds a local sparse hash-set to store its *k*-mers. For each process, thread *T*_1 _loads the reads from disk and transfers the reads to thread *T*_0 _through the message queue, where the short reads are arranged into batches and a message contains a batch of reads. *T*_0 _receives batches of reads from the queue, calculates the hash values of all *k*-mers, and stores some of them in its local sparse hash-set depending on the hash values. The cooperation of the two threads overlaps the computation and the file I/O operations, thus reducing the execution time. Since a *k*-mer and its reverse complement are considered equivalent in a *k*-molecule, we only need to store the canonical *k*-mer into the sparse hash-set to represent the *k*-molecule.

For any read containing non-A/C/G/T bases, PASHA converts those non-A/C/G/T bases to the base "A" (as Velvet does), not simply discarding the whole read as ABySS does [9]. After completing the generation of *k*-mers, each process writes its local *k*-mers to disk for future use when constructing the preliminary de Bruijn graph. Our distributed *k*-mer generation implementation can also be used (directly or after modification) by other tools, such as CUDA_EC [15] and Quake [16] to generate and count the occurrences of *k*-mers in genomic data.

### Distributed de Bruijn graph construction

The preceding stage only generates *k*-mers, and does not record any graph-related information for a *k*-mer. However, to construct a de Bruijn graph, we need not only the *k*-mers themselves, but also multiplicity and linkage information. A common approach is to use a hash-map implementation, using *k*-mers as keys, to provide fast access to the graph-related information. However, this approach will result in a large memory overhead. Conway and Bromage [17] suggested a sparse bitmap data structure to represent the de Bruijn graph, achieving memory efficiency at the cost of execution time. However, their proof-of-concept assembler (functionally similar to our Stages (i) and (ii) ) takes about 50 hours and yields a highly fragmented assembly, with an N50 *contig *size of only 250, for the Yoruban male genome. Hence, we exclude it from the following assessments. In PASHA, we instead use a sorted vector data structure to store the *k*-mers and their graph-related information. Each process loads its local *k*-mers from disk and stores them in a sorted vector. The sorted vector is sorted using the *k*-mers as keys.

We use the same approach as ABySS to represent the linkage information between nodes; i.e., the linkages are compacted into 8 bits with each bit representing the presence or absence of each of the eight edges in the two directions. However, to build the linkages between nodes, PASHA employs a different approach. For each *k*-mer (each node), ABySS checks the existence of all possible neighbours by doing all possible base extensions in each direction. If a neighbour exists, it sets its corresponding bit to represent the existence of the linkage. This approach is effective but has a probability of introducing spurious edges, which connect *k*-mers that are not adjacent in any read. Hence, PASHA builds linkages directly from the adjacency information of *k*-mers in the input reads, i.e., a linkage between two *k*-mers is created if and only if the two *k*-mers are adjacent in at least one read.

**Figure 2:**
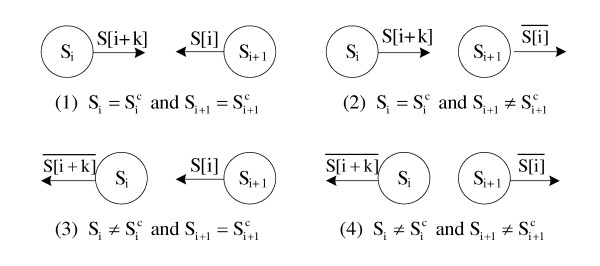
**Linkage construction for two adjacent k-mers in a read**.

While building linkages, for each process, thread *T*_1 _loads batches of reads from disk and transfers them to *T*_0 _as the previous stage does. For each read *S*, *T*_0 _iterates over each *k*-mer *S*_*i *_and identifies its location after calculating the base-4 representation of its canonical *k*-mer. If the *k*-mer belongs to it, *T*_0 _sets the corresponding linkage bits calculated from the bases *S*[*i*-1] and *S*[*i*+*k*], which are the extension bases in the left and the right directions of *S*_*i*_. When the index is out of the range, the corresponding extension base (and its complement) is an invalid base Ø, indicating that no linkage is created in that direction. Figure 2 shows all four cases of the linkage construction between two adjacent *k*-mers in a read. Because each process iterates all *k*-mers in all input reads, no communication between processes is required during the construction process. While constructing the linkages, we compute the multiplicity of each *k*-mer at the same time. In PASHA, two bytes are used to represent the multiplicity of a *k*-mer. After completing the linkage construction, we will get a distributed preliminary de Bruijn graph with each node corresponding to a *k*-mer.

### Graph simplification

The preliminary de Bruijn graph contains many linear chains of nodes that can be merged to simplify the graph without loss of information. We start the simplification from the removal of spurious linkages. Generally, there are three major kinds of spurious linkages: *tips*, *low-coverage paths *and *bubbles*. A *tip *is a short and low-coverage dead end, which is likely to be caused by sequence errors at the beginning or the end of reads. A *low-coverage path *only covers one or a few reads and is likely to be a chimeric connection. *Bubbles *are redundant paths with minor differences, which might be due to heterozygosity, internal read errors or nearby tips connecting. At this stage, we only remove tips and low-coverage paths, leaving the removal of bubbles to the following stage.

Prior to the removal of longer tips, we first remove low-frequency dead-end individual *k*-mers. This removal relies on the assumption that the majority of true *k*-mers should occur in several reads, i.e. the multiplicity of a true *k*-mer is supposed to be above a minimum multiplicity threshold *M*, which is automatically estimated from the multiplicities of all *k*-mers. This removal work is conducted round-by-round until no dead-end *k*-mers meet the removal conditions. For each process, *T*_0 _identifies the *k*-mers, which are dead-end and have a multiplicity less than *M*, in its local collection of *k*-mers, and then removes the *k*-mers and their linkages in the graph. When removing linkages to *k*-mers in another process, *T*_0 _packs a request message and lets *T*_1 _forward to the remote process. *T*_1 _forwards the request from *T*_0 _to other processes, and handles the requests on the removal of the specific linkages to its local *k*-mers, from the other processes.

In PASHA, we simply remove tips that are shorter than 2*k*. For each process, *T*_0 _generates the current linear chain of *k*-mers, starting from a dead-end *k*-mer, by extending the chain in the left or right directions base-by-base. If the chain is longer than 2*k*, the chain is released; and otherwise, the *k*-mers in the chain will be removed as well as their linkages. If, during the extension of a chain, a linked *k*-mer exists locally, *T*_0 _gets the graph-related information of the *k*-mer directly from its local sorted vector. Otherwise, *T*_0 _packs a request message and lets *T*_1 _forward it to the remote process. *T*_0 _will be blocked until receiving the response, forwarded back by *T*_1_, from the remote process.

After completing the removal of tips, the graph is split into different linear chains of nodes. All processes cooperate in parallel to generate the sequences corresponding to the linear chains. The linear chains are generated using two steps. The first step generates linear chains starting from dead-end *k*-mers, where a chain is extended in only one direction. The second step starts from an arbitrary *k*-mer, where a chain must be extended in two directions. In the first step each process *P*_*i *_extends a linear chain from each active local dead-end *k*-mer until another dead-end *k*-mer is met. In this case, *P*_*i *_checks the location process *P*_*j *_of the dead-end *k*-mer to avoid duplicates because *P*_*j *_(if *i *≠ *j*) might be generating this linear chain at the same time. In our algorithm, *P*_*i *_keeps this linear chain only if *P*_*i *_≤ *P*_*j*_, and releases it, otherwise. This process will be conducted iteratively until there are no local dead-end *k*-mers in each process. The second step is completed by assigning processes one-by-one to generate linear chains. At any time, only one process *P*_*i *_is allowed to generate linear chains and the other processes have to wait and process requests from *P*_*i*_. *P*_*i *_starts the two-directional extension from each local *k*-mer until a loop or a dead-end *k*-mer is found. In this case, the loop is simply broken up and output as a linear chain. For each sequence, the coverage is calculated by dividing the sum of multiplicities of the *k*-mers in its corresponding chain by the sequence length. If the coverage is lower than the minimum coverage threshold, the sequence should be given up since it is likely to be generated from linear chains containing spurious connections. The remaining sequences are written to disk for the use in the next stage.

### Bubble merging and contig generation

This stage consists of three steps. First, a Velvet-like de Bruijn graph is constructed from the sequences produced from the previous stage. To build the Velvet-like graph, we form a node, as well as its twin node, from a sequence and create linkages between nodes by aligning reads to nodes. If two adjacent *k*-mers in a read belong to two different nodes, we create an edge connecting them if there is no edge between them, and otherwise, update the information of the existing edge. While aligning reads to nodes, we do not record any mapping information about the reads. We employ a multi-threaded implementation to accelerate the alignment of reads to nodes on multi-core CPUs, where a single read is aligned to the graph nodes by a thread and locks are carefully employed to guarantee the mutual exclusive access to critical sections (e.g. the creation and updating of links between nodes).

Secondly, we detect and merge bubbles in the graph. The "Tour-bus" method in Velvet is employed to detect bubbles. The detected bubbles are merged into a single path if the sequences of the parallel paths meet the user-specified similarity requirement. In PASHA, we directly use the source code of the "Tour-bus" method, but modified the conditions to merge the two paths. Two paths are merged if they have at most a two-base-pair difference in length with ≥ 90% identity.

Finally, we simplify the graph after further removal of short tips and low-coverage nodes, and then generate *contigs *from nodes.

### Scaffolding

The scaffolding work aims to find the correct ordering of the assembled *contigs*, and then joins them into *scaffolds*. The determination of the ordering of *contigs *relies on the mapping information of paired-end reads onto the *contigs*, and then the mapping information is transferred to scaffolding linkages between *contigs*.

In PASHA, the scaffolding work starts from the construction of a Velvet-like de Bruijn graph from the assembled *contigs*. While aligning paired-end reads to the graph nodes (we extend the *k*-mer based alignment algorithm in Velvet to provide support for multi-threading), the mapping information of a read pair, such as mapping locations and node identifiers, is recorded into an in-disk database if the two reads successfully map onto the graph. A similar multi-threaded design, as in the previous stage, is employed to accelerate the graph construction on multi-core CPUs.

Having completed the read mapping, the median insert size, as well as its standard deviation, for each library is estimated from the mapping information of paired-end reads whose two reads map onto the same nodes. We employ a modified Pebble algorithm [13] to do the scaffolding work. The scaffolding linkages are built from the mapping information of paired-end reads in the in-disk database. Velvet constructs linkages from the mapping information of reads and read pairs. For a single read, if it overlaps with more than one node, linkages will be created between these nodes. For a read pair, both of which have overlaps with nodes, a linkage is created between two nodes that respectively have overlaps with the two reads. In PASHA, we only use the mapping information of read pairs to construct linkages, where a linkage is considered reliable if we have at least three read pairs to form the linkage.

### Speed optimizations

In the representation of the preliminary de Bruijn graph, PASHA employs a sorted vector data structure, instead of a hash-map, to store *k*-mers and their graph-related information. While reducing memory overhead, a sorted vector causes an increase in the average search time of *k*-mers. Given *N k*-mers stored in a sorted vector, the average search time is about log*N*, generally longer than the (nearly) constant search time of a hash-map. In this case, we build an acceleration table using the most significant *r *bits (*r *= 24 by default) of a *k*-mer to speed up the search. This acceleration table only results in a memory increase of 2^*r *^times the size of type integer bytes, but is expected to reduce the average search time to log(*N*/2^*r*^).

For Stages (i) and (ii), each MPI process has two threads *T*_0 _and *T*_1_: one for communication and the other for local computation. This mechanism is expected to improve the execution speed by overlapping communication and computation. However, when the two threads communicate frequently, along with memory allocations and de-allocations at the same time, the overhead incurred by system calls on memory operations may offset the performance obtained from the overlapping. Thus, we use the *tbb_allocator *template class in the Intel Threading Building Blocks library to manage the memory allocation and de-allocation for the communication between *T*_0 _and *T*_1_. The *tbb_allocator *template class does improve the execution speed through its smart management of user memory allocation and de-allocation.

## Results and Discussion

### Experimental data

To assess PASHA, we use four paired-end short read datasets from four different genomes to conduct experiments (see Table 1). The first three datasets: *Bacillus*, *Bordetella *and *E.coli *have accession numbers DRR000002, ERR007648 and SRR001665 in the NCBI Sequence Read Archive (SRA), respectively. The reference genome of the *Bacillus *dataset is *Bacillus subtilis subsp. subtilis str. 168 *with accession number NC_000964 in GenBank; the reference genome of the *Bordetella *dataset is *Bordetella pertussis Tohama I *with accession number NC_002929; and the reference genome of the *E.coli *dataset is *Escherichia coli str. K-12 substr. MG1655 *with accession number NC_000913. The Yoruban male dataset has the accession number SRA000271 in NCBI SRA, which contains six sub-datasets with accession numbers SRX000600, SRX000601, SRX000602, SRX000603, SRX001539 and SRX001540, respectively. The first four sub-datasets come from the same library CT1194 and the last two sub-datasets from library CT1373. We have used the six sub-datasets (about 4.05 billion reads) to produce *contigs *and used the first four sub-datasets to create *scaffolds *(about 3.76 billion 200bp-insert-size paired-end reads). The use of these short read datasets is consistent for both PASHA and ABySS to produce *contigs *or *scaffolds*.

**Table 1:** Short read datasets for assembler assessment

	Bacillus	Bordetella	E.coli	Yoruban male
library	160bp	198bp*	200bp	200bp
read length	36	36	36	36~42
no. of reads	16,633,474	12,549,138	20,816,448	3,758,659,514
coverage	142×	111×	162×	44×
genome size	4,215,606	4,086,189	4,639,675	3,101,788,170**

* uses an estimated insert size from assembly due to the unavailability of the real library insert size; ** uses the total length of all scaffolds in the GRCh37/hg19 build human reference sequence.

### Assembly quality assessment

We have assessed the assembly quality of PASHA by comparing it to three leading assemblers: Velvet (version 1.0.17), ABySS (version 1.2.1) and SOAPdenovo (version 1.04) using the datasets in Table 1. All the tests are conducted on a workstation with two quad-core 2.40 GHz CPUs and 72 GB memory, and on a compute cluster with 8 compute nodes connected by a high-speed Infiniband switch. Each node of the cluster consists of two quad-core 2.93 GHz CPUs and 24 GB memory, running the Linux operating system.

The assembly quality of all assemblers is compared in terms of NG50, NG80 and maximum *contig *or *scaffold *sizes. The NG50 (NG80) *contig *or *scaffold *size is calculated by ordering all assembled sequences by their lengths, and then adding the lengths from the largest to the smallest until the summed length exceeds 50% (80%) of the reference genome size. In this paper, for each dataset, we use the same reference genome size (shown in Table 1) to calculate the NG50 (NG80) *contig *or *scaffold *size for all assemblers. This is different from the calculation used in the ABySS and SOAPdenovo papers, where they consider the total length of all assembled sequences by each assembler as the reference genome size. For the calculation of *scaffold *sizes, the intra-scaffold gaps are included. For the calculation of genome coverage, we split the *scaffolds *into their constituent *contigs *at the position of gaps that are filled by a series of "N" bases. The genome coverage and the number of incorrect *contigs *are computed from the results obtained from aligning *contigs *to their reference genomes using BLAT version 34 [18]. A *contig *is considered correct if it has a full length alignment to the reference genome with a minimum identity of 95% (the number of matches dividing the *contig *length) and a maximal error rate of 5%. The alignment length is calculated by summing up the number of matches, the number of mismatches, and the number of insertions in the query and the target. The error rate is calculated by dividing the sum of the number of mismatches and the number of insertions in the query and the target by the alignment length.

We first use the three small paired-end datasets (i.e. *Bacillus*, *Bordetella *and *E.coli*) to evaluate the different assemblers in terms of assembly quality and execution speed on a single CPU core of the workstation. The parameters of all the assemblers have been carefully tuned with the intention to gain the highest assembly quality for each dataset, where each assembler chooses the *k*-mer size that produces the largest NG50 *scaffold *size. Table 2, 3 and 4 show the assembly results of all four assemblers, where we only consider *scaffolds *of length ≥ 100 bps. The tables show that for all the three datasets, PASHA is able to produce more contiguous assemblies with comparable genome coverage and mis-assembly rates in terms of all measures relating to *scaffolds*. Moreover, PASHA achieves the fastest execution speed on a single CPU core. PASHA uses a single MPI process (having two threads) for the first two stages of the pipeline, and a single thread for the last two stages. However, due to the small sizes of datasets, thread *T*_1 _contributes little to the actual execution time by overlapping file I/O operations with *T*_0_.

**Table 2:** Assembly results for *Bacillus*

	PASHA	Velvet	ABySS	SOAPdenovo
no. of scaffolds	20	80	66	98
NG50	1,435,675	670,481	424,309	487,364
NG80	182,534	117,643	124,700	96,291
max	2,044,786	919,263	890,628	918,694
mean	208,124	52,046	67,457	42,399
genome coverage	92.27%	98.69%	97.92%	97.60%
incorrect contigs (mean)	5(61,643)	1(44,055)	1(70,485)	2(22,680)
time (in seconds)	332	433	747	467

PASHA uses the parameters "k = 29", Velvet uses "k = 29, -exp_cov = auto, -cov_cutoff = auto", ABySS uses "k = 29, n = 10" and SOAPdenvo uses "k = 23, insert_length = 160".

**Table 3:** Assembly results for *Bordetella*

	PASHA	Velvet	ABySS	SOAPdenovo
no. of scaffolds	228	294	287	298
NG50	24,517	18,063	18,150	17,870
NG80	10,006	8,237	9,215	8,157
max	121,801	75,085	75,809	74,881
mean	16,508	12,797	13,520	12,583
genome coverage	70.44%	68.45%	53.67%	72.45%
incorrect contigs (mean)	166(5,521)	150(6,834)	138(12,172)	81(10,261)
time (in seconds)	207	292	484	293

PASHA uses the parameters "k = 31", Velvet uses "k = 31, -exp_cov = auto, -cov_cutoff = auto", ABySS uses "k = 31, n = 10" and SOAPdenvo uses "k = 25, insert_length = 198".

**Table 4:** Assembly results for *E.coli*

	PASHA	Velvet	ABySS	SOAPdenovo
no. of scaffolds	64	179	124	166
NG50	164,390	95,486	96,308	105,781
NG80	63,677	43,814	43,972	41,901
max	297,975	268,283	268,372	221,692
mean	71,305	25,465	37,381	27,406
genome coverage	97.44%	98.67%	95.58%	97.97%
incorrect contigs (mean)	8(6,145)	5(9,909)	5(39,765)	8(7,202)
time (in seconds)	325	490	595	533

PASHA uses the parameters "k = 31", Velvet uses "k = 31, -exp_cov = auto, -cov_cutoff = auto", ABySS uses "k = 33, n = 10" and SOAPdenvo uses "k = 23, insert_length = 215".

To demonstrate the capability of PASHA to handle large genomes, we have further assembled the genome of a Yoruban male individual using the above compute resources. The first two stages of PASHA are computed on the 8-node cluster, and the last two stages run on the 8-core workstation. The *contig *generation of ABySS is computed on the 8-node cluster and the scaffolding is performed on the 8-core workstation. Both PAHSA and ABySS use a *k*-mer size of 27 for the assembly. Tables 5 and 6 show the PASHA and ABySS assembly results both with, and without, scaffolding between PASHA and ABySS, where we only consider *contigs *and *scaffolds *of lengths ≥ 100bps. Without scaffolding, PASHA produces an NG50 *contig *size of 503 with a largest *contig *length of 18,981 and ABySS gives an NG50 *contig *size of 513 with a largest *contig *length 15,909. As for genome coverage, PASHA correctly aligned 98.88% of the *contigs*, covering about 66.47% of the human genome, and ABySS correctly aligned 99.18% of the *contigs*, covering about 68.90% of the human genome. The longest correct *contig *has a length of 18,252 for PASHA, indicating that the largest *contig *failed to be aligned to the human genome, and a length of 15,909 for ABySS. With scaffolding, PASHA yields an NG50 *scaffold *size of 2,294 and a largest *scaffold *length of 54,491, giving a genome coverage of about 66.94%. ABySS gives an NG50 *scaffold *size of 1,326 and a largest *scaffold *length of 29,862, giving a genome coverage of about 71.52%. Overall, PASHA demonstrates competitive assembly quality with ABySS in terms of *contigs *and *scaffolds*.

**Table 5:** Assembly results for the Yoruban male genome without scaffolding

	PASHA	ABySS
NG50	503	513
max	18,981	15,909
mean	581	543
median	283	261
genome coverage	66.47%	68.90%
no. of contigs	3,518,718	3,916,628
incorrect contigs (mean)	39,419(467)	31,189(413)
sum (bps)	2,045,433,773	2,125,482,148

**Table 6:** Assembly results for the Yoruban male genome with scaffolding

	PASHA	ABySS
NG50	2,294	1,326
max	54,491	29,862
mean	1,948	1,170
median	973	636
genome coverage	66.94%	71.52%
no. of scaffolds	1,133,810	1,893,930
incorrect contigs (mean)	70,160(367)	27,367(726)
sum (bps)	2,208,249,938	2,216,254,604

In terms of execution speed, PASHA takes about 21 hours to complete the whole assembly, running on 32 cores (i.e. using 16 MPI processes) in the 8-node cluster with 24 GB per node (a total memory size of 192 GB) and on the 8-core workstation with 72 GB memory (details are shown in Table 7). Using the same compute resources, ABySS takes about 50.6 hours, about 2.38× slower than PASHA. Hence, we may say that PASHA has a higher performance-cost ratio than ABySS for the assembly of genomes as large as the human genome.

**Table 7:** Runtime of PASHA and utilized compute resources for different stages

Stages	Time (h)	No. of CPUs
*k*-mer generation and distribution	0.7	32
de Bruijn graph construction and simplification	3.1	32
bubble merging and contig generation	11.6	8
scaffolding	5.9	8
overall	21.3	N/A

### Scalability

To evaluate the scalability of PASHA, we have carried out the assemblies of the three small datasets on a different number of CPU cores. The first two stages of PASHA are executed on the compute cluster using different numbers of CPU cores, and the last two stages on a single node using four threads. Since PASHA uses two threads for one MPI process, we start the evaluation from two CPU cores.

**Figure 3:**
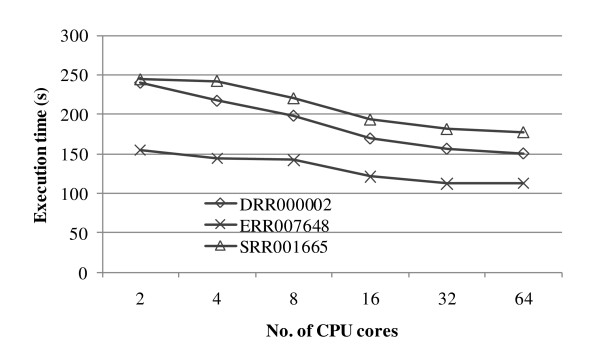
**Execution time of PASHA on different numbers of CPU cores**.

Figure 3 shows the execution time of PASHA on different numbers of CPU cores. From the figure, it can be seen that PASHA is able to decrease the execution time as the number of CPU cores increases. However, limited by the execution time of the last two stages, the overall execution time decreases slowly and will ultimately reach a plateau. Furthermore, we have compared the execution speed between PASHA and ABySS using the same number of CPU cores in the above cluster. Using the three datasets, PASHA is about 2.25× faster on average than ABySS. We did not compare the execution time with SOAPdenovo since it is a multi-threaded algorithm for shared-memory systems and requires a very large amount of memory for the human genome assembly problem.

## Conclusions

In this paper, we have presented PASHA, a parallelized short read assembler for large genomes using de Bruijn graphs. Taking advantage of both shared-memory multi-core CPUs and distributed-memory compute clusters, PASHA has demonstrated its potential to perform high-quality de-novo assembly of large genomes in reasonable time with modest compute resources.

Our evaluation using three small real paired-end datasets shows that PASHA is able to produce better assemblies with comparable genome coverage and mis-assembly rates compared to three leading assemblers: Velvet, ABySS and SOAPdenovo. Moreover, PASHA achieves the fastest speed for all three datasets on a single CPU. For the Yoruban male genome, PASHA is able to complete the assembly in about 21 hours with modest compute resources, which is about 2.38× faster than ABySS running on the same compute resources. Without scaffolding, PASHA yields an NG50 *contig *size of 503 with the longest correct *contig *length of 18,252, and with scaffolding, it produces an NG50 *scaffold *size of 2,294. PASHA achieves competitive assembly quality with ABySS, but takes less execution time using the same compute resources. For scalability, PASHA is able to reduce the execution time as the number of CPU cores increases, and is about 2.25× faster on average than ABySS running on the same number of CPU cores.

## Abbreviations

CPU: Central Processing Unit; MPI: Message Passing Interface; SRA: Sequence Read Archive

## Authors' contributions

YL conceptualized the study, carried out the design and implementation of the algorithm, performed benchmark tests, analyzed the results and drafted the manuscript; BS conceptualized the study, participated in the algorithm optimization and analysis of the results and contributed to the revising of the manuscript; DLM conceptualized the study, participated in the analysis of the results, and contributed to the revising of the manuscript. All authors read and approved the final manuscript.

## References

[B1] Warren RL Sutton GG Jones SJ Holt RA Assembling millions of short DNA sequences using SSAKE Bioinformatics 2007 23 4 500 501 10.1093/bioinformatics/btl629 17158514 PMC7109930

[B2] Jeck WR Reinhardt JA Baltrus DA Hickenbotham MT Magrini V Mardis ER Dangl JL Jones CD Extending assembly of short DNA sequences to handle error Bioinformatics 2007 23 21 2942 2944 10.1093/bioinformatics/btm451 17893086

[B3] Dohm JC Lottaz C Borodina T Himmelbauer H SHARCGS, a fast and highly accurate short-read assembly algorithm for de novo genomic sequencing Genome Res 2007 17 11 1697 1706 10.1101/gr.6435207 17908823 PMC2045152

[B4] Schmidt B Sinha R Beresford-Smith B Puglisi SJ A fast hybrid short read fragment assembly algorithm Bioinformatics 2009 25 17 2279 2280 10.1093/bioinformatics/btp374 19535537

[B5] Ariyaratne PN Sung WK PE-Assembler: de novo assembler using short paired-end reads Bioinformatics 2011 27 2 167 174 10.1093/bioinformatics/btq626 21149345

[B6] Pevzner PA Tang H Waterman MS An Eulerian path approach to DNA fragment assembly Proc Natl Acad Sci USA 2001 98 17 9748 9753 10.1073/pnas.171285098 11504945 PMC55524

[B7] Butler J MacCallum I Kleber M Shlyakhter IA Belmonte MK Lander ES Nusbaum C Jaffe DB ALLPATHS: de novo assembly of whole-genome shotgun microreads Genome Res 2008 18 5 810 820 10.1101/gr.7337908 18340039 PMC2336810

[B8] Zerbino DR Birney E Velvet: algorithms for de novo short read assembly using de Bruijn graphs Genome Res 2008 18 5 821 829 10.1101/gr.074492.107 18349386 PMC2336801

[B9] Simpson JT Wong K Jackman SD Schein JE Jones SJ Birol I ABySS: a parallel assembler for short read sequence data Genome Res 2009 19 6 1117 1123 10.1101/gr.089532.108 19251739 PMC2694472

[B10] Li R Zhu H Ruan J Qian W Fang X Shi Z Li Y Li S Shan G Kristiansen K Li S Yang H Wang J Wang J De novo assembly of human genomes with massively parallel short read sequencing Genome Res 2010 20 2 265 272 10.1101/gr.097261.109 20019144 PMC2813482

[B11] Gnerre S Maccallum I Przybylski D Ribeiro FJ Burton JN Walker BJ Sharpe T Hall G Shea TP Sykes S Berlin AM Aird D Costello M Daza R Williams L Nicol R Gnirke A Nusbaum C Lander ES Jaffe DB High-quality draft assemblies of mammalian genomes from massively parallel sequence data Proc Natl Acad Sci USA 2011 108 4 1513 1518 10.1073/pnas.1017351108 21187386 PMC3029755

[B12] Jackson BG Regennitter M Yang X Schnable PS Aluru S Parallel de novo assembly of large genomes from high-throughput short reads25th IEEE International Symposium on Parallel & Distributed Processing (IPDPS) 2010 1 10

[B13] Zerbino DR McEwen GK Margulies EH Birney E Pebble and Rock Band: heuristic resolution of repeats and scaffolding in the Velvet short-read de novo assembler PLoS One 2009 4 12 e8407 10.1371/journal.pone.0008407 20027311 PMC2793427

[B14] Bentley DR Balasubramanian S Swerdlow HP Smith GP Milton J Brown CG Hall KP Evers DJ Barnes CL Bignell HR Boutell JM Bryant J Carter RJ Keira Cheetham R Cox AJ Ellis DJ Flatbush MR Gormley NA Humphray SJ Irving LJ Karbelashvili MS Kirk SM Li H Liu X Maisinger KS Murray LJ Obradovic B Ost T Parkinson ML Pratt MR Accurate whole human genome sequencing using reversible terminator chemistry Nature 2008 456 7218 53 59 10.1038/nature07517 18987734 PMC2581791

[B15] Shi H Schmidt B Liu W Müller-Wittig W A parallel algorithm for error correction in high-throughput short-read data on CUDA-enabled graphics hardware J Comput Biol 2010 17 4 603 615 10.1089/cmb.2009.0062 20426693

[B16] Kelley DR Schatz MC Salzberg SL Quake: quality-aware detection and correction of sequencing errors Genome Biol 2010 11 11 R116 21114842 10.1186/gb-2010-11-11-r116 PMC3156955

[B17] Conway TC Bromage AJ Succinct data structures for assembling large genomes Bioinformatics 2011 27 4 479 486 10.1093/bioinformatics/btq697 21245053

[B18] Kent WJ BLAT--the BLAST-like alignment tool Genome Res 2002 12 656 664 11932250 10.1101/gr.229202 PMC187518

